# Long-term economic effect of telecare on patients with chronic diseases

**DOI:** 10.1186/1472-6963-14-S2-P128

**Published:** 2014-07-07

**Authors:** Masatsugu Tsuji, Yuji Akematsu

**Affiliations:** 1 University of Hyogo, Kobe, Japan; 2 Osaka University, Toyonaka, Japan

## Background

Although it is often observed that telecare (e-Health) contributes to the health of the elderly or patients at home, it is difficult to obtain scientifically rigorous evidence to support this. This study aims to examine the long-term effect of telecare implementation on the number of treatment days and the medical expenditure to users with chronic diseases including heart diseases, hypertension, stroke, and diabetes in a project of Nishi-aizu Town, Fukushima Prefecture, Japan. The data covers from 2002-2010. The town’s telecare system is used to remotely monitor users’ health by transmitting health data, such as blood pressure, blood oxygen level, and ECG, to the town’s Health Center.

## Materials and methods

The method of analysis is to compare the above outcomes of two groups, namely users (treatment) and non-users (control) of the system based on the receipt data issued by National Health Insurance. Data analyzed is from 2002-2010 on the outcomes for 91 users and 118 non-users. Panel data analysis, in particular the generalized least squares with random-effect model was conducted. The cross-term such as user dummy multiplied by one of chronic diseases was introduced in the estimation equation to examine how telecare affects to users with chronic diseases.

## Results

The results obtained are as follows: regarding the estimation of treatment days, only heart diseases and hypertension were significant; the treatment days of users who had heart diseases were smaller than non-users by 6.6 days per year (p<0.001), and that of users with hypertension were smaller by 2.0 days (p<0.001). As for medical expenditure, heart diseases and hypertension were significant; the medical expenditure of users with hypertension was smaller by JPY 58,766 (USD 588) per year (p<0.01), and that of users with hypertension was smaller by JPY 21,272 (USD 213) (p<0.01).

## Conclusions

This study demonstrates the long-term effect of telecare. The results of the authors’ previous studies based on five-year data from 2002-2006 of this town showed that the amount of reduction in medical expenditure related to users with heart diseases and hypertension was about JPY 39,081 (USD 391) and JPY 21,859 (USD 219), respectively. These show that the longer telecare is used, the more medical expenditure is reduced for users with these diseases. The amount of reduction in medical expenditure is larger than the annual operational costs of this project, which amounts to about JPY 60,000 (USD 600) per user. This project thus satisfies sustainability criteria.

